# Genome-Wide Analysis of Rad52 Foci Reveals Diverse Mechanisms Impacting Recombination

**DOI:** 10.1371/journal.pgen.0030228

**Published:** 2007-12-14

**Authors:** David Alvaro, Michael Lisby, Rodney Rothstein

**Affiliations:** 1 Department of Genetics and Development, Columbia University Medical Center, New York, New York, United States of America; 2 Department of Molecular Biology, University of Copenhagen, Copenhagen, Denmark; Brandeis University, United States of America

## Abstract

To investigate the DNA damage response, we undertook a genome-wide study in Saccharomyces cerevisiae and identified 86 gene deletions that lead to increased levels of spontaneous Rad52 foci in proliferating diploid cells. More than half of the genes are conserved across species ranging from yeast to humans. Along with genes involved in DNA replication, repair, and chromatin remodeling, we found 22 previously uncharacterized open reading frames. Analysis of recombination rates and synthetic genetic interactions with *rad52*Δ suggests that multiple mechanisms are responsible for elevated levels of spontaneous Rad52 foci, including increased production of recombinogenic lesions, sister chromatid recombination defects, and improper focus assembly/disassembly. Our cell biological approach demonstrates the diversity of processes that converge on homologous recombination, protect against spontaneous DNA damage, and facilitate efficient repair.

## Author Summary

Homologous recombination (HR) is a cellular process that permits efficient repair of both endogenous and exogenous DNA damage. Although the principal players in HR have been well characterized, the interplay of diverse processes with the HR pathway remains mysterious. Traditionally, genetic screens investigating HR have utilized genetic assays, such as survival following exposure to DNA damaging agents or alterations in the rate of the generation of recombinant products. In this work, we instead utilize a cell biology phenotype, the relocalization of the central HR protein Rad52 into subnuclear foci reflecting repair centers actively engaged in HR. This approach allows us to identify mutants that affect the kinetics of HR repair center assembly and disassembly regardless of the outcome of recombination. We identified 86 gene deletions that lead to increases in the levels of spontaneous foci in proliferating diploid cells, 22 of which were deletions of previously uncharacterized ORFs (designated *IRC2–11*, *13–16*, *18–25*). Genetic characterization of the mutants revealed a diversity of mechanisms that underlie the focus phenotype. These include increasing the generation of DNA lesions, blocking the completion of HR, and altering the kinetics of genetic recombination and the assembly/disassembly of the HR protein complexes.

## Introduction

Homologous recombination (HR), a repair mechanism that depends on DNA sequence homology, underlies a number of important DNA processes that act to both stabilize and diversify a genome. In mitotic cells, HR functions to maintain the integrity of the genome through such processes as the repair of DNA double-strand breaks (DSBs), the maintenance of rDNA copy number, and the rescue of collapsed replication forks. HR is entirely conservative when it occurs following DNA replication where a sister chromatid is available as a template. However, utilization of sequences on a homologous chromosome can lead to crossovers and potential loss of heterozygosity (LOH), while recombination at ectopic or repeated sequences may lead to genomic rearrangements such as deletions, duplications, and translocations (reviewed in [1]).

In Saccharomyces cerevisiae, Rad52 is the defining member of an epistasis group that includes: RecA homologs Rad51, Rad55, Rad57, and Dmc1; putative SWI/SNF family ATPase Rad54; Rad52 homolog Rad59 and Mre11, Xrs2, and Rad50. The Rad52 epistasis group is essential for HR. Rad52 binds single-stranded DNA in vitro and has been shown to stimulate DNA annealing and to enhance Rad51-catalyzed strand invasion [2–5].

In response to DNA damage, proteins involved in HR relocalize into discrete subnuclear foci. Fluorescently tagged repair and checkpoint proteins have been used to explore the composition and dynamics of these foci, which are giga-dalton-sized assemblies of proteins [6]. Repair foci colocalize with fluorescently tagged inducible DSB sites, regions of single-stranded DNA, and sites of unscheduled DNA synthesis [7–9]. Multiple DSBs often colocalize at a single focus showing that foci reflect recombination centers capable of the simultaneous repair of more than one lesion. The assembly of proteins into repair foci is a coordinated process beginning with detection of damage by the Mre11/Rad50/Xrs2 complex. Next, checkpoint proteins are bound and activated to arrest cell cycle progression until completion of repair. The lesion is repaired through HR performed by the Rad52 epistasis group proteins and finally the repair apparatus is disassembled [10]. From a cell biology perspective, Rad52 focus formation is an excellent marker for HR, since it is required for the recruitment of all other HR proteins into repair foci.

While exogenous DNA damage greatly stimulates the formation of Rad52 foci, foci also form spontaneously in S phase cells, likely reflecting the repair of spontaneous DNA lesions such as DSBs, nicks, and single-stranded gaps [6,11]. Time-lapse microscopy indicates that foci form in approximately 50% of cells during S phase and most spontaneous foci persist for less than 10 min [7]. Since spontaneous foci generally last for only a fraction of S phase, they are observed in 20% of S phase cells in a population of logarithmically growing cells (5% of the total population). Mutants defective in various aspects of DNA metabolism, including damage checkpoints (*mec1 sml1*), HR (*rad51*Δ), and DNA replication (*pol12–100*) exhibit elevated levels of spontaneous foci [6]. This elevation may be the consequence of an increased incidence of focus formation reflecting the generation of more DNA lesions, or the consequence of foci that persist over time resulting from an alteration in the dynamics of focus assembly/disassembly.

Here we report the results of a genome-wide screen designed to identify gene deletions that significantly alter levels of spontaneous Rad52 foci. The set of gene deletions identified includes many known genes involved in DNA and chromatin processes such as replication, repair, silencing, and chromosome segregation, as well as a number of other processes with no reported link to HR. In addition, 22 previously uncharacterized ORFs designated *IRC2–11*, *13–16*, *18–25* (Increased Recombination Centers) were identified. Measurement of HR between sister chromatids and between homologous chromosomes in these focus mutants established four different classes demonstrating that several distinct mechanisms are involved in precipitating increased Rad52 foci. Furthermore, several *IRC* genes exhibit synthetic interactions with a *rad52*Δ allele suggesting a direct role in the maintenance of genomic integrity.

## Results

### A Genome-Wide Screen to Identify New Genes Affecting DNA Metabolism

**Figure 1:**
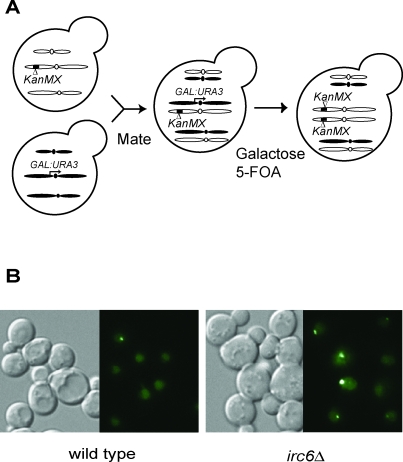
Rad52-YFP Focus Screen Using Systematic Hybrid LOH (A) Method for preparation of hybrid LOH strains for screening, permitting simultaneous complementation of recessive factors in the library strains and introduction of the Rad52-YFP plasmid through mating [12]. *KanMX*-marked gene deletion strains are mated with corresponding conditional centromere strains. Growth in galactose medium drives transcription through the centromere destabilizing the chromosome and 5-FOA selects for cells that have lost the conditional chromosome. In most cases, loss is followed by endoduplication of the monosomic chromosome, generating a 2n hybrid diploid homozygous for the gene deletion. (B) Spontaneous Rad52-YFP foci in a wild-type strain and in an *irc6*Δ deletion, a strain identified in the screen with an increased fraction of cells containing spontaneous foci.

Rad52, a central recombination protein, relocalizes to form sub-nuclear foci in response to spontaneous and induced DNA damage. Therefore, Rad52 focus levels can be used as a sensitive indicator for processes that impinge on the genome. An initial screen to identify gene deletions affecting the levels of spontaneous Rad52 foci was performed by transforming a plasmid containing a Rad52-YFP fusion gene directly into library haploid strains. However, this approach yielded an excessive number of false-positive results caused by additional recessive factors in the individual library strains. We therefore developed a method that permits the systematic creation of hybrid diploids that are homozygous for the gene deletion from each library strain (Figure 1A), while simultaneously facilitating the introduction of plasmid or chromosomal constructs into the gene deletion strains [12].

**Figure 2:**
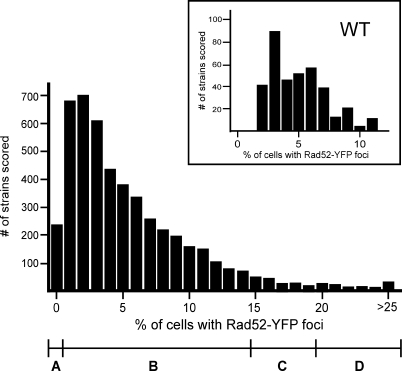
Distribution of 4,805 Library Gene Deletions for Rad52-YFP Focus Levels The levels are the percentage of cells with one or more spontaneous Rad52-YFP foci among 200–400 homozygous hybrid diploid cells screened. The strains were divided into four subsets based upon focus levels: (A) zero foci (4.8% of the library); (B) 0.1%–15% foci (90% of the library); (C) 15%–20% foci (3.0% of the library); and (D) >20% foci (2.2% of the library). The inset represents the distribution of 354 repetitions of the wild-type parent strain BY4742 scored concurrently with the gene deletions during the screen.

Using the systematic hybrid LOH method, we screened 4,805 nonessential gene deletions for levels of spontaneous foci (Figure 1B). Twenty gene deletions could not be constructed as hybrid homozygous diploids, including 11 that are deficient in mating. The distribution of focus levels is shown in Figure 2. To evaluate the reliability of the mutant screen, we partitioned the genes into four sub-sets shown (A, B, C, and D). Sub-set B consists of the strains that exhibit focus levels within the range of variation seen in a wild-type strain. These account for approximately 90% of the deletions screened. For 233 gene deletions (sub-set A), no foci were observed. Upon retest, most of these strains formed foci; however, the distribution was shifted slightly to the left compared to the entire deletion library (Figures 2 and S1A). Furthermore, for the 96 strains that showed the lowest levels upon retest (less than 1% foci), all gave rise to foci after ionizing radiation, indicating that no fundamental aspect of Rad52 focus formation was disrupted in these deletion strains (Figure S1B). Further study of this group of genes may shed some light on processes that generate spontaneous DNA damage.

We next examined those mutants that exhibit foci in more than 20% of all cells (sub-set D), a 4-fold elevation over average wild-type levels. These 108 gene deletions (2.2% of the library) fall into a number of broad functional groups involved in various aspects of DNA and chromatin metabolism. Following two independent retests, 80 mutants (75% of the 108) consistently exhibited elevated focus levels including deletions of 16 previously uncharacterized ORFs (Tables 1 and S1D).

**Table 1:**
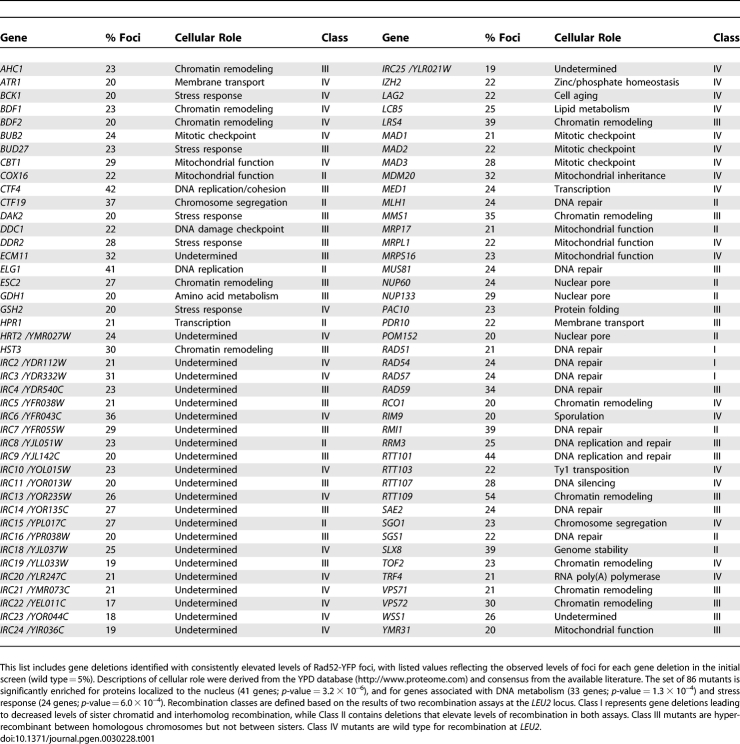
Genes Identified in the Screen for Increased Spontaneous Rad52-YFP Foci

Subsequently, we examined the 144 deletions from the genome-wide screen that exhibited foci in 15%–20% of cells (sub-set C). We suspected that interesting candidate genes would be found in this sub-set since it includes deletions of several genes directly associated with genomic integrity, including replication (*CTF18*, *RAD27*), homologous repair (*MRE11*, *RAD50*), chromatin remodeling (*HPA1*, *SET2*, *SNT1*, *SWI6*), and cell cycle control (*CLB5*, *RAD17*). To decide how much further to study this sub-set, we retested all 41 uncharacterized ORFs contained within it. We found that only nine (22%) consistently exhibited elevated focus levels upon retesting (Table S1C). Since only 22% of uncharacterized ORFs in this 15%–20% focus range maintained an elevated focus phenotype compared to 75% in the greater than 20% range, we concluded that the likelihood of identifying additional new genes in this sub-set was dramatically decreased. Therefore, we did not delve deeper into our candidate pool, and the nine new genes along with the 80 deletions exhibiting the highest levels of foci were selected for further analysis (Table 1).

Lastly, we recognize that the screen produces some false negatives since several gene deletions expected to exhibit elevated focus levels (e.g., *mre11*Δ, *xrs2*Δ, *rad50*Δ, *rad27*Δ) failed the cutoff for significance (16%, 13%, 9%, and 16%, respectively). In addition, the screen identified some gene deletions (e.g*.*, *ddc1*Δ, *rad57*Δ, *slx8*Δ, *mms1*Δ, and *rtt109*Δ), but failed to identify other members of the same complexes (e.g*.*, *mec3*Δ and *rad17*Δ, *rad55*Δ, *slx5*Δ, *asf1*Δ, and *mms22*Δ) that were expected to be phenotypically equivalent. We then retested the other members individually and they consistently demonstrated elevated levels of spontaneous foci (Table S1E) indicating that the focus phenotype was shared among all members of each complex and that the mutants not identified in the initial screen were false negatives. We did not include these mutants in our subsequent analysis, as we studied only those gene deletions identified by our original screening criteria.

### *IRC* Genes Are Assigned to 22 Uncharacterized ORFs

Among the 89 deletions that exhibit elevated levels of spontaneous Rad52 foci were 25 previously uncharacterized ORFs. Eight of these *IRC* genes are listed as dubious ORFs on the *Saccharomyces* Genome Database due to the small size of the coding region. In addition, three *IRC* ORFs overlap other genes identified in the screen (*IRC1* and *BDF2*, *IRC15* and *CTF19*, *IRC17* and *RTT103*), while several others overlap genes not identified in the screen. To verify that the focus phenotype observed in each *IRC* mutant was the result of disruption of that ORF, we performed a complementation test. Twenty-two of the 25 *ircΔ*s were complemented by their corresponding ORF. However, a wild-type copy of the *IRC* ORF was unable to complement *irc1*Δ (*ydl071c*Δ), *irc12Δ *(*yor024w*Δ), and *irc17*Δ (*ydr290w*Δ) (Figure S2A). Of these three, *irc1*Δ and *irc17*Δ remove sequences from overlapping genes that were also identified in the screen and their focus phenotype was complemented by a plasmid containing the neighboring gene (Figures S2B and S2D). For *irc12Δ* the adjacent non-overlapping gene *HST3* complemented the phenotype (Figure S2C). We conclude that the elevated focus phenotype in these three strains was likely a consequence of mutation of the neighboring genes or regulatory sequences, thus reducing the number of mutant strains to 86.

### Defining Genetic Interaction Sub-Networks

Increased levels of Rad52 foci may indicate a dependency on *RAD52* gene function. In fact, 14 of the 86 mutants previously showed a synthetic fitness defect or lethality when combined with a *rad52*Δ allele [13–19]. Synthetic genetic interactions between all 86 gene deletions and *rad52*Δ were assayed through a comprehensive quantitative analysis (see Materials and Methods). The analysis revealed 27 synergistic interactions, 15 of which had not been previously described. All interactions observed resulted in synthetic growth defects, with no double mutants exhibiting improved growth compared to the single mutants. One gene deletion, *nup60*Δ, exhibited synthetic lethality with the *rad52*Δ allele. To distinguish between strong and weaker interactions, the remaining 26 were parsed into synergistic and additive growth defects (see Materials and Methods). Growth differences revealed 17 synergistic and 10 additive interactions (Table 2). Finally, we are unable to reproduce the previously reported synthetic interactions of *mus81*Δ and *rtt107*Δ with the *rad52*Δ allele [18]. The 27 interactions described here suggest that the absence of these genes intensifies the requirement for HR in cell survival and growth.

**Table 2:**
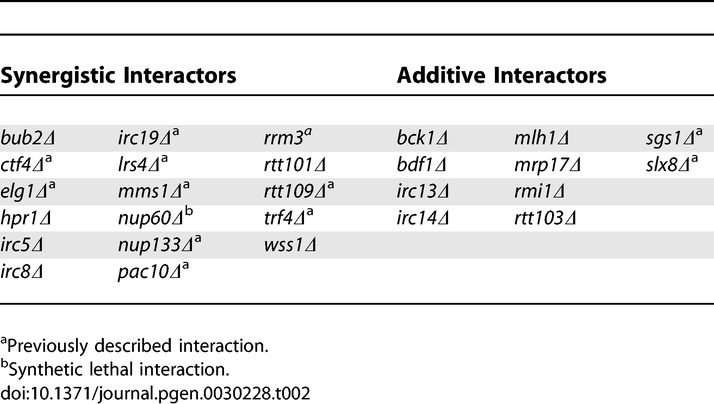
Mutants with *rad52*Δ Synthetic Interactions

### Mitotic Recombination Rates Define Four Mutant Classes

**Figure 3:**
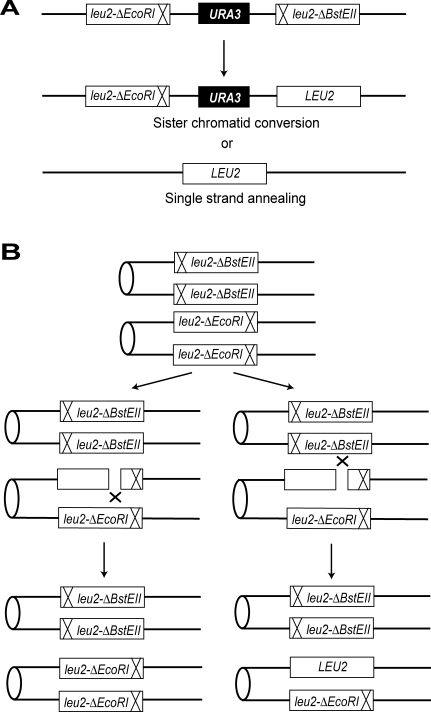
Recombination Assays Used in This Study (A) The direct repeat recombination assay measures intra-chromosomal and sister chromatid recombination events that generate a functional *LEU2* allele from two *leu2* heteroalleles. The *URA3* marker between the heteroalleles permits discrimination between sister chromatid conversion events (Leu+ Ura+ recombinants) and SSA (Leu+ Ura− recombinants). (B) The interhomolog recombination assay measures recombination between *leu2* heteroalleles on homologous chromosomes in diploid cells. Chromosomes are shown following DNA synthesis and reflect a pair of sister chromatids for each chromosome homolog. A recombinogenic lesion occurring at the *LEU2* locus is most often repaired using the template on the sister chromatid, which is a conservative event resulting in no loss or gain of genetic information (left). However, a gene conversion event or reciprocal exchange between two homologous chromosomes can generate a functional *LEU2* allele (right).

The mutants were parsed into functional classes based on their effect on homologous recombination. The increase in spontaneous Rad52 foci observed in the mutant strains may correspond to altered levels of spontaneous recombination. This hypothesis is underscored by the observation that a number of these mutants have been previously demonstrated to exhibit either elevated (*hpr1*Δ) or reduced (*rad51*Δ) mitotic recombination [20,21]. Each focus mutant was subjected to two heteroallelic recombination assays to sort them into functional classes corresponding to the mechanisms that lead to the accumulation of Rad52 foci (Figure 3). The direct repeat assay measures the rate of sister chromatid gene conversion and intrachromosomal single-strand annealing (SSA) events, while the interhomolog assay measures recombination between alleles on homologous chromosomes in diploids, specifically those recombination events that do not utilize the sister chromatid, which is the preferred template for HR [22]. Interhomolog recombination between heteroalleles is one underlying cause of LOH.

The recombination rates for the two assays divide the set of mutants into four classes (Tables 1, 3, and 4). The three Class I mutants are in the *RAD52* epistasis group and exhibit significantly reduced levels of gene conversion between both sister chromatids and homologous chromosomes but wild-type levels of SSA, consistent with previous studies [21,23]. The 14 gene deletions that exhibit increased rates of both direct repeat and interhomolog recombination define Class II. The 32 mutants in Class III exhibit increased rates of recombination specifically between homologous chromosomes, while sister chromatid recombination (SCR) in the direct repeat assay is unaffected. Surprisingly, 37 Class IV gene deletions fail to demonstrate any alteration in the rate of recombination with either assay. It is possible that these mutants affect recombination only at specific genomic regions. To begin to examine this notion, we tested 33 of the Class IV deletion strains using an assay that measures recombination in the multiple tandem rDNA array [24]. Elevated frequencies of recombination in the rDNA array compared to wild type (1.7 × 10^−2^ ± 0.7) are observed in four gene deletion strains (*bck1*Δ 18 ×10^−2^ ± 2.2, *bdf1*Δ 7.4 ×10^−2^ ± 2.9, *rtt107*Δ 5.8 × 10^−2^ ± 2.3, and *trf4*Δ 9.3 × 10^−2^ ± 3.7), demonstrating that the focus phenotype may be triggered by events at specific loci.

**Table 3:**
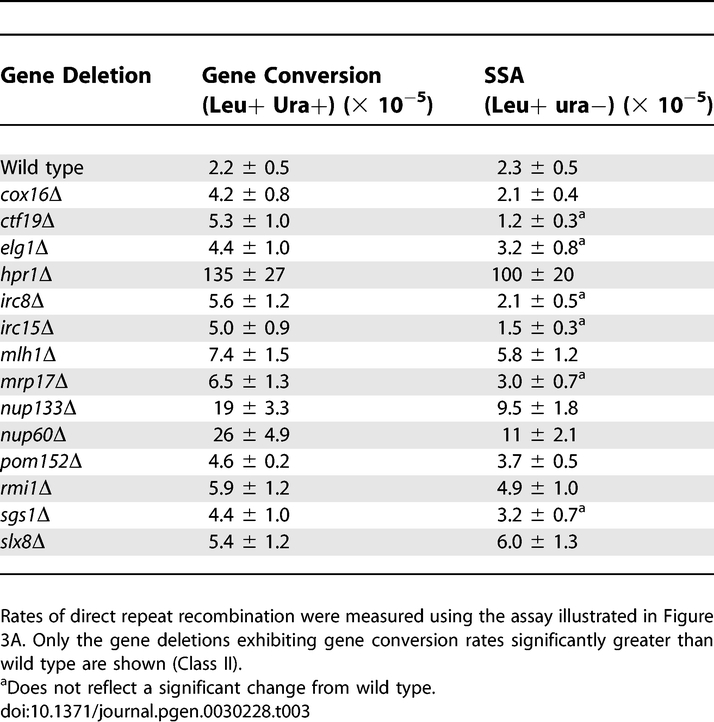
Direct Repeat Recombination Rates

**Table 4:**
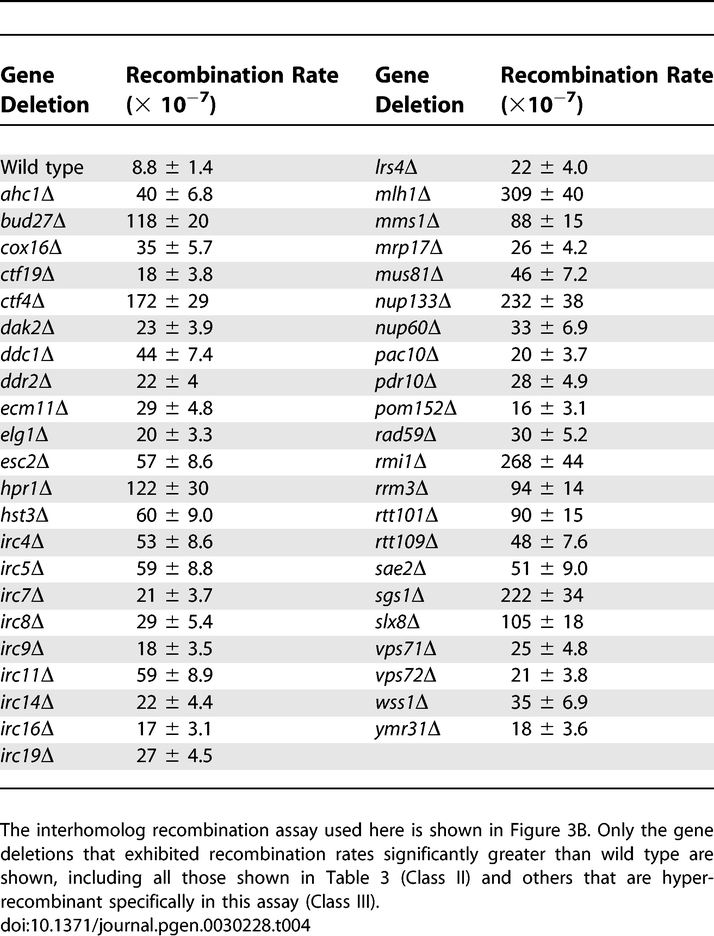
Interhomolog Recombination Rates

## Discussion

To identify pathways involved in the maintenance of genomic integrity, we used a newly developed method, systematic hybrid loss of heterozygosity, to introduce a cell biological assay into over 4,800 nonessential gene deletion strains. The levels of spontaneous relocalization of Rad52-YFP protein into subnuclear foci were analyzed in individual gene deletion strains. Our screen differs from previous genomic studies where changes in DNA metabolism phenotypes were identified using the output of DNA repair or recombination assays, such as survival following exposure to exogenous DNA damage or the gain or loss of genetic markers following recombination. Our cell biological screen permits the identification of alterations in the HR pathway in living cells regardless of the outcome of these events. In addition to mutations affecting recombination that ultimately block the appearance of recombinants (Class I) and mutations that increase the formation of spontaneous DNA damage (Class II), our screen uncovered mutations that alter the kinetics rather than the frequency of events (Class III) along with those that do not affect recombination globally (Class IV).

The majority of the genes identified in this study fall into functional groups associated with DNA metabolism and chromosome dynamics, including replication and repair, transcription, and chromatin remodeling (Table 1). In addition, we found genes involved in nuclear pore complexes and mitochondrial function as well as diverse cellular processes such as the spindle assembly checkpoint not previously associated with HR. Finally, one quarter of the genes identified in our screen (22) were uncharacterized. It is notable that many of these *IRC* genes are very small ORFs, which would have reduced the likelihood of identifying them in traditional mutagenesis screens. Indeed, most have either no apparent impact on the outcome of recombination (Class IV) or a subtle effect below the sensitivity of other assays (Class III).

**Figure 4:**
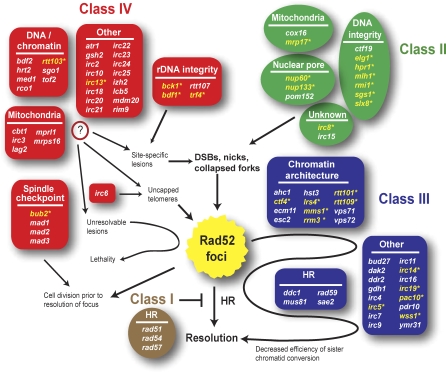
Pathways Leading to Increased Rad52 Foci Are Revealed by This Study Class I mutants block HR subsequent to the formation of Rad52 foci. Class II mutants stimulate the formation of DNA lesions, which is reflected as increased focus formation and results in increased sister chromatid and interhomolog recombination. Class III mutants decrease the efficiency of sister chromatid recombination through effects on cohesion, chromatin architecture, HR, and other mechanisms, thereby increasing the duration of Rad52 foci (indicated by the meandering line). Class IV mutants do not have a global effect on homologous recombination. However, the focus phenotype in some mutants reflects recombination at specific sites such as rDNA or telomeres. Other mutants may cause the division of cells prior to resolution of HR foci, or lead to the generation of lesions that cannot be repaired, resulting in lethality. For all classes, mutations with an asterisk and highlighted in yellow, sensitize cells to the absence of Rad52 (see Table 2).

Both direct repeat and interhomolog recombination assays were used to parse the complete set of mutants into a handful of classes that indicate the mechanisms leading to the focus phenotype. The four classes of mutants were based upon their recombination phenotype: Class I: decreased Rad51-dependent HR; Class II: increased HR between sister chromatids and between homologous chromosomes; Class III: increased HR specifically between homologous chromosomes; and Class IV: wild-type levels of HR (Figure 4). The quantitative analysis of synthetic genetic interactions between all 86 mutants and *rad52*Δ provide further insight into the subdivisions within the classes.

### Class I: Hypo-Recombination

Three members of the *RAD52* epistasis group, *rad51*Δ, *rad54*Δ, and *rad57*Δ display elevated levels of Rad52-YFP foci and decreased levels of Rad51-dependent recombination (this study and [25]). Rad51, Rad54, and Rad57 are recruited to a DNA lesion subsequent to Rad52 focus formation and their recruitment is dependent on Rad52 [25]. Deletion of genes that encode proteins that function downstream of Rad52 in HR leads to a failure to complete the recombination process. Thus, the increased focus levels in these mutants likely reflect the persistence of Rad52-YFP foci. Since SSA is dependent upon Rad52, but not Rad51 or other later HR proteins, the rate of that process is not affected by these gene deletions [21,26]. Class I gene deletions do not exhibit synthetic interactions with *rad52*Δ, since they are in the same epistasis group. It is noteworthy that none of the newly defined *IRC* gene deletions fall into this class, indicating that these genes are not required for Rad51-dependent HR.

### Class II: Hyper-Recombination

Class II mutants exhibit elevated levels of recombination between sister chromatids and between homologous chromosomes likely reflecting an increase in the generation of spontaneous DNA lesions requiring repair via HR. Thus, the Class II focus phenotype reflects an increase in the overall frequency of formation of Rad52-YFP foci in these mutants. This class includes a number of genes with well-characterized roles in the maintenance of genomic integrity and the suppression of spontaneous DNA damage (*SGS1*, *RMI1*, *ELG1*, *MLH1*, *HPR1*, and *SLX8*), as well as all three nuclear pore genes identified in the study (*NUP60*, *NUP133*, and *POM152*) [27–35]. The Class II recombination phenotype is exhibited by two mitochondrial genes (*COX16* and *MRPI17*), which suggests that an increase in oxidative damage (e.g., reactive oxygen species) stimulates spontaneous DNA lesions in these mutants [36–38]. Among the Class II mutants, ten of 14, including the previously uncharacterized gene *IRC8*, sensitize cells to the absence of a functional *RAD52* allele. Such a synthetic defect is consistent with an increased requirement for Rad52-mediated HR in these gene deletion strains as a result of the increased generation of spontaneous lesions.

### Class III: Interhomolog recombination

Class III mutants exhibit elevated levels of recombination between homologous chromosomes in diploid cells, but wild-type levels of SCR in haploids. It is unlikely that the Rad52 focus phenotype observed in this class reflects a general increase in endogenous DNA damage, but rather that spontaneous lesions that do arise are processed differently in these mutants. In diploid cells, lesions are preferentially repaired using the sister chromatid as template, effectively preventing LOH that can result from repair from the homologous chromosome. In Class III mutants, the 2- to 20-fold elevation in interhomolog recombination occurs without a concomitant increase in SCR. We suggest that the increased utilization of the homolog for repair reflects a defect in the efficiency of SCR. However, reduced SCR in these mutants is not a result of a defect in sister chromatid cohesion since only one Class III mutant, *ctf4*Δ, exhibited precocious sister chromatid separation ([39] and unpublished data).

The increase in interhomolog recombination observed in Class III mutants may reflect a defect in the recombination center itself. Null mutants of members of the Mre11-Rad50-Xrs2 complex and certain separation-of-function alleles of *rad52* exhibit elevated levels of spontaneous Rad52-YFP foci and increased interhomolog recombination, identical to the phenotype of our Class III [11,40]. Class III includes three genes (*DDC1*, *RAD59*, and *SAE2*) that encode proteins that themselves localize to repair foci in response to DNA damage [25]. In addition, Mus81 protein functions downstream of Rad52 in the resolution of recombination intermediates [41,42]. Similar to Class I, these four mutants do not exhibit synthetic growth interactions with *rad52*Δ as they function in the same pathway.

Class III also includes deletions of a large number of genes with roles in chromatin modification and remodeling (*AHC1*, *ESC2*, *LRS4*, *RRM3*, *VPS71-VPS72*, *MMS1-RTT101-RTT109*, and *HST3*) [20,43–49]*.* Modified chromatin at the site of damage may itself function as a scaffold for the recruitment and assembly of the repair machinery. Remodeling tightly condensed chromatin is critical to allow repair proteins access to both the damaged DNA and the homologous template [50,51]. Perhaps chromatin defects delay use of the sister chromatid as the repair template. The delay, observed here as persistent Rad52 foci, may increase the use of the homologous chromosome for repair.

Since the majority of the previously characterized Class III genes function in DNA replication, repair, and chromatin dynamics, it is likely that the six newly identified *IRC* genes in this class (*IRC4*, *IRC5*, *IRC7*, *IRC9*, *IRC14*, and *IRC19*) are also involved in DNA metabolism. Like six of the 12 Class III genes implicated in chromatin metabolism described above, *irc5*Δ, *irc14*Δ, and *irc19*Δ exhibit synthetic growth defects with *rad52*Δ, suggesting related roles for these genes. The *irc19*Δ mutant also exhibits synthetic defects with other genes associated with the maintenance of genome integrity including genes involved in replication and HR [18]. *IRC5* encodes a putative Snf2 family DNA helicase with homology to the mammalian lymphoid-specific helicase HELLS, supporting a role in chromatin remodeling [46,52]. Genetic interactions of the *irc5*Δ mutant correlate strongly with those of replicative proteins (*rad27*Δ, *elg1*Δ, *rnh201*Δ, *pol30–79*, *rfc4-DAMP*, *rfc5-DAMP*), suggesting that Irc5 protein remodeling activity may be involved in DNA replication (N. Krogan, personal communication).

### Class IV: Mutants without Recombination Phenotype

Class IV gene deletions do not affect recombination specifically at the *LEU2* locus. Since it is possible that recombination is affected in other regions of the genome, we measured recombination rates within the rDNA multiple tandem array. We chose to examine this array next because it is a highly organized region where recombination is tightly regulated [53]. We identified four Class IV mutants (*bck1*Δ, *bdf1*Δ, *rtt107*Δ, and *trf4*Δ) that exhibit rDNA hyper-recombination. Similar to most of the mutants in Class II, three of the four (*bck1*Δ, *bdf1*Δ, and *trf4*Δ) show synthetic interactions with *rad52*Δ that is consistent with their potential roles in the suppression of genetic instability within this array. It is tempting to speculate that *IRC13*, *BUB2*, and *RTT103,* the three other Class IV genes that show synthetic interactions with *rad52*Δ may also be involved in the suppression of region-specific damage. Other regions of the genome that require specific factors for maintenance are telomeres. For example, defects in telomere capping may lead to increased recognition of telomeres as DSB ends that would recruit Rad52. Interestingly, *irc6*Δ may have such a defect (W. Zhang and D. Durocher, personal communication).

Alternatively, increased levels of Rad52 foci may occur without measurable effects on the products of recombination. For example, the focus phenotype may be due to slowing of the HR process or delaying the disassembly of foci without changing the outcome. In addition, a gene deletion may generate unrepairable lesions that could result in the accumulation of repair proteins into foci but lead to cell lethality. For the spindle assembly checkpoint mutants, *mad1*Δ, *mad2*Δ, *mad3*Δ, and *bub2*Δ, an increase in the number of cells with foci in G1 was observed (unpublished data). Mitotic division before resolution of a repair focus formed during the previous round of DNA replication could result in two G1 cells with potentially unresolvable foci [54,55]. The unique phenotype of the Class IV mutants underscores the utility of taking a cell biological approach to investigate HR. Examination of intermediate steps in the process permits the identification of genes that would not have been found using assays requiring a measurable recombination product.

The large number of chromatin remodeling, mitochondrial, and spindle checkpoint genes found in Class IV suggests that some of the 12 *IRC* genes in this class may be involved in these processes. For example, *IRC3* encodes a putative DEAD/DEAH box helicase that localizes to mitochondria and exhibits synthetic lethal interactions with deletions of spindle assembly checkpoint proteins, histones, and proteins associated with sister chromatin cohesion. Furthermore, *IRC20* encodes a putative Snf2/Swi2 family helicase, which localizes to nuclei and mitochondria and is implicated in transcriptional regulation, while *IRC21* is predicted to function in chromatin remodeling [14,38,46,56–58] (G. Prelich, personal communication).

### Evolutionary Conservation

Overall, the 86 genes identified in the Rad52-YFP focus screen are largely conserved throughout eukaryotic evolution, with 49 having homologs in nearly every sequenced eukaryotic species (Table S4). Homologs for another 15 have been found in evolutionarily divergent yeast species including Schizosaccharomyces pombe and Candida albicans, but not in mammals, Drosophila melanogaster, or Caenorhabditis elegans. The remaining 22 are found in the closely related sensu stricto yeast species. Seven *IRC* genes have homologs identified across eukaryota. This includes three putative helicases, *IRC3*, *IRC5*, and *IRC20.* Other *IRC* homologs have been linked to human diseases, including the *IRC7* homolog CTH, which encodes a cystathionase implicated in premature births and cancers, and the *IRC24* homolog SPR, which contains polymorphisms associated with Parkinson disease [59–61]. *IRC21* has homology to NADPH cytochrome B5 oxidoreductase, which is linked to insulin-dependent diabetes in mice, while *IRC15* resembles mammalian mitochondrial dihydrolipoamide dehydrogenase, associated with a number of human diseases including Alzheimer disease [62,63].

### Summary

The Rad52-YFP focus screen described here applies systematic hybrid LOH in a genome-wide cell biological search to identify proteins involved in diverse pathways contributing to genomic integrity. This assay permitted the identification of a large set of gene deletions that affect the incidence and dynamics of HR foci in living cells regardless of the genetic outcome. In particular, we uncovered 22 previously uncharacterized ORFs, many having only subtle effects on the process of recombination, which prevented their identification in other screens. Since recombination is a multi-step process, it will be of interest to examine the dynamics of other HR factors within our mutant set and the genome as a whole. Additionally, it will be important to show that the conserved genes in other organisms play similar roles in this process.

## Materials and Methods

### Strains and plasmids.

Individual deletions of nonessential genes made in the BY4742 and BY4739 *MAT*α strains were obtained from the *Saccharomyces* Gene Deletion Project [64]. The conditional chromosome strains used to create the hybrid LOH strains and the method of inducing LOH in these strains have been described [12]. Briefly, the systematic hybrid LOH method utilizes a set of 16 strains, each containing a conditional centromere construct on one of the 16 yeast chromosomes. Mating each gene deletion strain to the appropriate conditional centromere strain creates hybrid diploids that are transiently heterozygous for the gene deletion. After chromosome loss is induced for the conditional chromosome, homozygosis of the marked gene deletion occurs more than 95% of the time as a result of endoduplication of the monosomic chromosome. This method allows the introduction of any plasmid or chromosomal construct into individual mutants of the gene deletion library through mating rather than individual transformations.

Strain BY4742 was used as the wild-type control for the library background. Gene deletions from the library were backcrossed into the W303 background to create congenic strains. Gene deletion strains assayed for direct repeat recombination are congenic (minimum of four backcrosses) to W5880-3A (*MATα ADE2 leu2-*ΔEcoRI*::URA3::leu2-*ΔBstEII *TRP1 lys2*Δ *RAD5*). Homozygous diploid deletion strains tested for interhomolog recombination are congenic (minimum of five backcrosses) to W5309 (*MAT* **a** */α ADE2/ADE2 TRP1/trp1–1 LYS2/lys2*Δ *leu2-*ΔEcoRI*/leu2*-ΔBstEII). Deletion strains assayed for marker loss in the rDNA array are congenic (minimum of six backcrosses) to W6921-5A (*MATα ade2–1 can1–100 TRP1 LYS2 rDNA:ADE2-CAN1*)*.*

The pWJ1314 plasmid, which expresses Rad52-YFP from the native *RAD52* promoter, was used for all focus measurements performed using library gene deletion strains [12]. Plasmids used for complementation analysis of *irc* mutants were built utilizing plasmid pWJ1250, designed to facilitate cloning via HR in yeast. To construct plasmid pWJ1250, overlapping primers C-D-TOP (AGCGAGGTCGACTAGGGATAACAGGGTAATCCGCTGCTAGGCGCGCCGTGGTTAACGTCAG) and C-D-BOTTOM (CAGCTGGAGCTCTAGGGATAACAGGGTAATGCAGGGATGCGGCCGCTGACGTTAACCAC) were fused and amplified by PCR with primers C-D-Forward (AGCGAGGTCGACTAGG) and C-D-Reverse (CAGCTGGAGCTCTAGG). This reaction resulted in a synthetic DNA containing common adaptamer sequences C and D separated by the restriction site for the blunt-cutting HpaI enzyme. The C-D cassette is flanked by 17 base pair recognition sites for the I-SceI meganuclease, and then flanked by SacI and SalI sites. The amplified DNA was digested with SacI and SalI for cloning into the yeast shuttle vector pRS416 [65]. Thus, DNA amplified with C- and D-containing adaptamers can be cloned into the HpaI-linearized pWJ1250 by transformation into yeast. Cloned cassettes can be recovered by I-SceI digestion of the resulting plasmid.

For each *IRC* and adjacent gene cloned for complementation analysis, C- and D-containing adaptamer primers were selected 200–300 bp upstream and downstream of the ORF. PCR products for each gene were amplified from a wild-type yeast strain and cotransformed into yeast with HpaI-linearized pWJ1250. The resulting gap-repaired plasmids were recovered from Escherichia coli and transformed into the gene deletion strains congenic (minimum of six backcrosses) to W3749-14C (*MAT**a** ADE2 bar1::LEU2 trp1–1 LYS2 RAD52-YFP)* along with plasmid pWJ1250 as a control. Complementation was performed by comparing levels of spontaneous Rad52-YFP foci in each gene deletion strain when transformed with the vector containing the deleted gene and with the empty vector as a control (Figure S2).

In the focus screen described, 108 mutants were initially identified with Rad52-YFP foci in greater than 20% of cells examined. Of these 108, 80 gene deletions maintained the focus phenotype following repeat experiments and were selected for further analysis. After retesting all uncharacterized ORFs with focus levels between 15%–20% (41), nine consistently exhibited elevated foci and were added to the set of deletions. Three hypothetical ORFs were removed from the set after expression of the wild-type gene failed to complement the focus phenotype in the gene deletion strain. The remaining 86 gene deletions were prepared for the additional assays described below.

### Microscopy.

Examination of Rad52-YFP focus levels by microscopy was performed as previously described [66]. Briefly, cells were grown overnight in SC-Leu media at 23 °C and exponentially growing cultures were prepared for microscopy. A single DIC image and 11 YFP images obtained at 0.3-μm intervals along the *z-*axis were captured for each frame, and Rad52-YFP foci were counted by inspecting all focal planes intersecting each cell. For each gene deletion strain in the screen, 200–400 cells were scored for Rad52-YFP foci. All Rad52-YFP focus data are presented in Figures 2, S1, and S2, and Table S1.

### GO enrichment analysis.

We took advantage of the existing Gene Ontology (GO) annotations to determine whether our set of 86 mutants was enriched for any particular categories of genes. We utilized the Fisher's Exact Test to compute statistically significant enrichment of GO categories within the 86 mutants compared to those among the complete set of 4,805 mutants assayed for Rad52-YFP foci. The set of focus mutants exhibited enrichment in the GO component category for proteins localized to the nucleus (*p*-value = 3.2 × 10^−6^), as well as the GO biological process categories for DNA metabolism and response to stress (*p*-value = 1.3 × 10^−4^ and 6.0 ×10^−4^). The Bonferroni corrected threshold for significance among GO components was 2.0 × 10^−3^ and among GO processes was 1.5 × 10^−3^.

### Synthetic interactions.

Synthetic interactions between library gene deletions and *rad52Δ* were determined on the basis of spore colony size following tetrad dissection. In the initial analysis, all 86 library gene deletion strains were mated to W303 background strain W3777-17A (*MAT* **a** *ADE2 bar1::LEU2 trp1–1 LYS2 rad52::HIS5 RAD5*) and sporulated. Twenty-four tetrads were dissected for each cross, and dissection plates were scanned using Adobe Photoshop (Adobe Systems). Colony size was measured for each cross using a macro (Y. Deng, unpublished data) written for ImageJ software (W. Rasband, National Institutes of Health), followed by genotyping for segregating alleles. Average colony size and standard deviation were derived in each cross for the wild type, gene deletion, *rad52*Δ, and the double mutants, and normalized to the mean value for the wild-type segregants in the cross. For this study, we defined two different classes of synthetic interactions for growth. Additive interactions are defined when the average colony size for the double mutant is significantly less than the colony size of either single mutant. Synergistic interactions are defined when the average colony size of the double mutant is significantly less than the product of the normalized colony size values of either single mutant. The synthetic interactions reported here were verified in a more closely related W303 genetic background by performing a second trial with congenic deletion strains (minimum four backcrosses) and W3777-17A (*rad52*Δ). All data for synthetic interactions are presented in Table S2.

### Quantitation of mitotic recombination.

Spontaneous mitotic recombination between *leu2-*ΔEcoRI and *leu2-*ΔBstEII heteroalleles was measured between sister chromatids in haploid strains and between homologous chromosomes in diploid strains as previously described [67,68] (Figure 3). Rates of mitotic recombination were calculated as described by Lea and Coulson [69]. For each gene deletion mutant, eight independent trials were performed for each assay. Two-tailed *t* tests were applied to determine significant changes in recombination rates in gene deletion strains. Recombination rates measured for all mutants in both assays are presented in Table S3. Frequencies of marker loss in the rDNA array was determined using a modification of a described method [24].

## Supporting Information

Figure S1 Repeat Analysis of Strains Scored with Zero Rad52 Foci from the Initial Screen (A) Distribution of Rad52-YFP focus levels observed after a second trial for all 233 strains initially scored with zero foci. The resulting distribution is a slightly left-shifted version of the distribution for the entire library (Figure 1), demonstrating that most of these zero focus strains are false positives.
(B) Induced Rad52-YFP focus levels following 40 Gy of ionizing radiation observed for 96 strains with the lowest spontaneous focus levels from (A). The dashed line indicates the range of focus levels observed for ten wild-type strains screened following IR.
(59 KB PDF)

Figure S2 Complementation of the Rad52 Focus Phenotype in *IRC* Mutants (A) 25 *IRC* gene deletion strains containing a genomically integrated *RAD52-YFP* fusion gene were analyzed following the introduction of either a single copy empty vector (black bars) or a vector containing a full-length copy of the *IRC* ORF corresponding to the one deleted in the strain (white bars). Rad52-YFP levels were scored as in the initial screen. Twenty-two of 25 *IRC* genes complemented the focus phenotype, while the ORFs designated *IRC1*, *IRC12*, and *IRC17* failed to complement.
(B) The *irc1*Δ removes a portion of the *BDF2* gene, and introduction of a vector containing *BDF2* (which also contains the full-length copy of *IRC1*) complements the focus phenotype in an *irc1*Δ strain.
(C) The *IRC12* ORF is situated immediately between *HST3* and *AHC1,* two other genes that exhibit elevated Rad52-YFP foci when deleted, but does not overlap either gene. Introduction of a vector containing a full-length copy of *HST3* complements the focus phenotype in an *irc12*Δ strain, while *IRC12* and *AHC1* do not.
(D) The *irc17*Δ removes a portion of the *RTT103* gene, and introduction of a vector containing *RTT103* (which also contains the full-length copy of *IRC17*) complements the focus phenotype in an *irc17*Δ strain. Since the focus phenotype observed in *irc1*Δ, *irc12*Δ, and *irc17*Δ is complemented by an adjacent gene, the gene names were withdrawn from the *Saccharomyces* Genome Database.
(263 KB PDF)

Table S1 Rad52 Focus Screen Data (1.2 MB XLS)

Table S2 Rad52 Synthetic Interaction Data (62 KB XLS)

Table S3 Recombination Rates (27 KB XLS)

Table S4 Eukaryotic Homologs (41 KB XLS)

## Abbreviations

DSB: double-strand break
HR: homologous recombination
IRC: increased recombination center
LOH: loss of heterozygosity
SCR: sister chromatid recombination
SSA: single-strand annealing

## References

[pgen-0030228-b001] Lisby M, Rothstein R (2004). DNA damage checkpoint and repair centers. Curr Opin Cell Biol.

[pgen-0030228-b002] Sung P (1997). Function of yeast Rad52 protein as a mediator between replication protein A and the Rad51 recombinase. J Biol Chem.

[pgen-0030228-b003] New JH, Sugiyama T, Zaitseva E, Kowalczykowski SC (1998). Rad52 protein stimulates DNA strand exchange by Rad51 and replication protein A. Nature.

[pgen-0030228-b004] Song B, Sung P (2000). Functional interactions among yeast Rad51 recombinase, Rad52 mediator, and replication protein A in DNA strand exchange. J Biol Chem.

[pgen-0030228-b005] Stasiak AZ, Larquet E, Stasiak A, Muller S, Engel A (2000). The human Rad52 protein exists as a heptameric ring. Curr Biol.

[pgen-0030228-b006] Lisby M, Rothstein R, Mortensen UH (2001). Rad52 forms DNA repair and recombination centers during S phase. Proc Natl Acad Sci U S A.

[pgen-0030228-b007] Lisby M, Mortensen UH, Rothstein R (2003). Colocalization of multiple DNA double-strand breaks at a single Rad52 repair centre. Nat Cell Biol.

[pgen-0030228-b008] Raderschall E, Golub EI, Haaf T (1999). Nuclear foci of mammalian recombination proteins are located at single-stranded DNA regions formed after DNA damage. Proc Natl Acad Sci U S A.

[pgen-0030228-b009] Haaf T, Raderschall E, Reddy G, Ward DC, Radding CM (1999). Sequestration of mammalian Rad51-recombination protein into micronuclei. J Cell Biol.

[pgen-0030228-b010] Lisby M, Rothstein R (2005). Localization of checkpoint and repair proteins in eukaryotes. Biochimie.

[pgen-0030228-b011] Lettier G, Feng Q, de Mayolo AA, Erdeniz N, Reid RJ (2006). The role of DNA double-strand breaks in spontaneous homologous recombination in S. cerevisiae. PLoS Genet.

[pgen-0030228-b012] Alvaro D, Sunjevaric I, Reid RJ, Lisby M, Stillman DJ (2006). Systematic hybrid LOH: a new method to reduce false positives and negatives during screening of yeast gene deletion libraries. Yeast.

[pgen-0030228-b013] Loeillet S, Palancade B, Cartron M, Thierry A, Richard GF (2005). Genetic network interactions among replication, repair and nuclear pore deficiencies in yeast. DNA Repair (Amst).

[pgen-0030228-b014] Tong AH, Lesage G, Bader GD, Ding H, Xu H (2004). Global mapping of the yeast genetic interaction network. Science.

[pgen-0030228-b015] Kanellis P, Agyei R, Durocher D (2003). Elg1 forms an alternative PCNA-interacting RFC complex required to maintain genome stability. Curr Biol.

[pgen-0030228-b016] Bellaoui M, Chang M, Ou J, Xu H, Boone C (2003). Elg1 forms an alternative RFC complex important for DNA replication and genome integrity. EMBO J.

[pgen-0030228-b017] Torres JZ, Schnakenberg SL, Zakian VA (2004). Saccharomyces cerevisiae Rrm3p DNA helicase promotes genome integrity by preventing replication fork stalling: viability of *rrm3* cells requires the intra-S-phase checkpoint and fork restart activities. Mol Cell Biol.

[pgen-0030228-b018] Pan X, Ye P, Yuan DS, Wang X, Bader JS (2006). A DNA integrity network in the yeast Saccharomyces cerevisiae. Cell.

[pgen-0030228-b019] Kouprina N, Kroll E, Bannikov V, Bliskovsky V, Gizatullin R (1992). *CTF4 (CHL15)* mutants exhibit defective DNA metabolism in the yeast *Saccharomyces cerevisiae*. Mol Cell Biol.

[pgen-0030228-b020] Driscoll R, Hudson A, Jackson SP (2007). Yeast Rtt109 promotes genome stability by acetylating histone H3 on lysine 56. Science.

[pgen-0030228-b021] Ivanov EL, Sugawara N, Fishman-Lobell J, Haber JE (1996). Genetic requirements for the single-strand annealing pathway of double-strand break repair in Saccharomyces cerevisiae. Genetics.

[pgen-0030228-b022] Kadyk LC, Hartwell LH (1992). Sister chromatids are preferred over homologs as substrates for recombinational repair in Saccharomyces cerevisiae. Genetics.

[pgen-0030228-b023] Rattray AJ, Symington LS (1995). Multiple pathways for homologous recombination in Saccharomyces cerevisiae. Genetics.

[pgen-0030228-b024] Fritze CE, Verschueren K, Strich R, Easton Esposito R (1997). Direct evidence for *SIR2* modulation of chromatin structure in yeast rDNA. EMBO J.

[pgen-0030228-b025] Lisby M, Barlow JH, Burgess RC, Rothstein R (2004). Choreography of the DNA damage response: spatiotemporal relationships among checkpoint and repair proteins. Cell.

[pgen-0030228-b026] McDonald JP, Rothstein R (1994). Unrepaired heteroduplex DNA in Saccharomyces cerevisiae is decreased in *RAD1 RAD52*-independent recombination. Genetics.

[pgen-0030228-b027] Gangloff S, McDonald JP, Bendixen C, Arthur L, Rothstein R (1994). The yeast type I topoisomerase Top3 interacts with Sgs1, a DNA helicase homolog: a potential eukaryotic reverse gyrase. Mol Cell Biol.

[pgen-0030228-b028] Watt PM, Hickson ID, Borts RH, Louis EJ (1996). *SGS1*, a homologue of the Bloom's and Werner's syndrome genes, is required for maintenance of genome stability in Saccharomyces cerevisiae. Genetics.

[pgen-0030228-b029] Mullen JR, Nallaseth FS, Lan YQ, Slagle CE, Brill SJ (2005). Yeast Rmi1/Nce4 controls genome stability as a subunit of the Sgs1-Top3 complex. Mol Cell Biol.

[pgen-0030228-b030] Aroya SB, Kupiec M (2005). The Elg1 replication factor C-like complex: a novel guardian of genome stability. DNA Repair (Amst).

[pgen-0030228-b031] Kramer B, Kramer W, Williamson MS, Fogel S (1989). Heteroduplex DNA correction in Saccharomyces cerevisiae is mismatch specific and requires functional PMS genes. Mol Cell Biol.

[pgen-0030228-b032] Wellinger RE, Prado F, Aguilera A (2006). Replication fork progression is impaired by transcription in hyperrecombinant yeast cells lacking a functional THO complex. Mol Cell Biol.

[pgen-0030228-b033] Zhang C, Roberts TM, Yang J, Desai R, Brown GW (2006). Suppression of genomic instability by *SLX5* and *SLX8* in Saccharomyces cerevisiae. DNA Repair (Amst).

[pgen-0030228-b034] Burgess RC, Rahman S, Lisby M, Rothstein R, Zhao X (2007). The Slx5-Slx8 complex affects sumoylation of DNA repair proteins and negatively regulates recombination. Mol Cell Biol.

[pgen-0030228-b035] Rout MP, Aitchison JD, Suprapto A, Hjertaas K, Zhao Y (2000). The yeast nuclear pore complex: composition, architecture, and transport mechanism. J Cell Biol.

[pgen-0030228-b036] Carlson CG, Barrientos A, Tzagoloff A, Glerum DM (2003). *COX16* encodes a novel protein required for the assembly of cytochrome oxidase in Saccharomyces cerevisiae. J Biol Chem.

[pgen-0030228-b037] Cadet J, Berger M, Douki T, Ravanat JL (1997). Oxidative damage to DNA: formation, measurement, and biological significance. Rev Physiol Biochem Pharmacol.

[pgen-0030228-b038] Sickmann A, Reinders J, Wagner Y, Joppich C, Zahedi R (2003). The proteome of Saccharomyces cerevisiae mitochondria. Proc Natl Acad Sci U S A.

[pgen-0030228-b039] Hanna JS, Kroll ES, Lundblad V, Spencer FA (2001). *Saccharomyces cerevisiae CTF18* and *CTF4* are required for sister chromatid cohesion. Mol Cell Biol.

[pgen-0030228-b040] Bressan DA, Baxter BK, Petrini JH (1999). The Mre11-Rad50-Xrs2 protein complex facilitates homologous recombination-based double-strand break repair in Saccharomyces cerevisiae. Mol Cell Biol.

[pgen-0030228-b041] Kaliraman V, Mullen JR, Fricke WM, Bastin-Shanower SA, Brill SJ (2001). Functional overlap between Sgs1-Top3 and the Mms4-Mus81 endonuclease. Genes Dev.

[pgen-0030228-b042] Bastin-Shanower SA, Fricke WM, Mullen JR, Brill SJ (2003). The mechanism of Mus81-Mms4 cleavage site selection distinguishes it from the homologous endonuclease Rad1-Rad10. Mol Cell Biol.

[pgen-0030228-b043] Eberharter A, Sterner DE, Schieltz D, Hassan A, Yates JR (1999). The ADA complex is a distinct histone acetyltransferase complex in Saccharomyces cerevisiae. Mol Cell Biol.

[pgen-0030228-b044] Dhillon N, Kamakaka RT (2000). A histone variant, Htz1p, and a Sir1p-like protein, Esc2p, mediate silencing at *HMR*. Mol Cell.

[pgen-0030228-b045] Smith JS, Caputo E, Boeke JD (1999). A genetic screen for ribosomal DNA silencing defects identifies multiple DNA replication and chromatin-modulating factors. Mol Cell Biol.

[pgen-0030228-b046] Shiratori A, Shibata T, Arisawa M, Hanaoka F, Murakami Y (1999). Systematic identification, classification, and characterization of the open reading frames which encode novel helicase-related proteins in Saccharomyces cerevisiae by gene disruption and Northern analysis. Yeast.

[pgen-0030228-b047] Krogan NJ, Keogh MC, Datta N, Sawa C, Ryan OW (2003). A Snf2 family ATPase complex required for recruitment of the histone H2A variant Htz1. Mol Cell.

[pgen-0030228-b048] Collins SR, Miller KM, Maas NL, Roguev A, Fillingham J (2007). Functional dissection of protein complexes involved in yeast chromosome biology using a genetic interaction map. Nature.

[pgen-0030228-b049] Schneider J, Bajwa P, Johnson FC, Bhaumik SR, Shilatifard A (2006). Rtt109 is required for proper H3K56 acetylation: a chromatin mark associated with the elongating RNA polymerase II. J Biol Chem.

[pgen-0030228-b050] Gontijo AM, Green CM, Almouzni G (2003). Repairing DNA damage in chromatin. Biochimie.

[pgen-0030228-b051] Green CM, Almouzni G (2002). When repair meets chromatin. First in series on chromatin dynamics. EMBO Rep.

[pgen-0030228-b052] McCarroll SA, Murphy CT, Zou S, Pletcher SD, Chin CS (2004). Comparing genomic expression patterns across species identifies shared transcriptional profile in aging. Nat Genet.

[pgen-0030228-b053] Torres-Rosell J, Sunjevaric I, De Piccoli G, Sacher M, Eckert-Boulet N (2007). The Smc5-Smc6 complex and SUMO modification of Rad52 regulates recombinational repair at the ribosomal gene locus. Nat Cell Biol.

[pgen-0030228-b054] Millband DN, Campbell L, Hardwick KG (2002). The awesome power of multiple model systems: interpreting the complex nature of spindle checkpoint signaling. Trends Cell Biol.

[pgen-0030228-b055] Cleveland DW, Mao Y, Sullivan KF (2003). Centromeres and kinetochores: from epigenetics to mitotic checkpoint signaling. Cell.

[pgen-0030228-b056] de la Cruz J, Kressler D, Linder P (1999). Unwinding RNA in Saccharomyces cerevisiae: DEAD-box proteins and related families. Trends Biochem Sci.

[pgen-0030228-b057] Huh WK, Falvo JV, Gerke LC, Carroll AS, Howson RW (2003). Global analysis of protein localization in budding yeast. Nature.

[pgen-0030228-b058] Tanay A, Sharan R, Kupiec M, Shamir R (2004). Revealing modularity and organization in the yeast molecular network by integrated analysis of highly heterogeneous genomewide data. Proc Natl Acad Sci U S A.

[pgen-0030228-b059] Vina J, Vento M, Garcia-Sala F, Puertes IR, Gasco E (1995). L-cysteine and glutathione metabolism are impaired in premature infants due to cystathionase deficiency. Am J Clin Nutr.

[pgen-0030228-b060] Klein CE, Roberts B, Holcenberg J, Glode LM (1988). Cystathionine metabolism in neuroblastoma. Cancer.

[pgen-0030228-b061] Sharma M, Mueller JC, Zimprich A, Lichtner P, Hofer A (2006). The sepiapterin reductase gene region reveals association in the PARK3 locus: analysis of familial and sporadic Parkinson disease in European populations. J Med Genet.

[pgen-0030228-b062] Xie J, Zhu H, Larade K, Ladoux A, Seguritan A (2004). Absence of a reductase, NCB5OR, causes insulin-deficient diabetes. Proc Natl Acad Sci U S A.

[pgen-0030228-b063] Mastrogiacoma F, Lindsay JG, Bettendorff L, Rice J, Kish SJ (1996). Brain protein and alpha-ketoglutarate dehydrogenase complex activity in Alzheimer disease. Ann Neurol.

[pgen-0030228-b064] Brachmann CB, Davies A, Cost GJ, Caputo E, Li J (1998). Designer deletion strains derived from Saccharomyces cerevisiae S288C: a useful set of strains and plasmids for PCR-mediated gene disruption and other applications. Yeast.

[pgen-0030228-b065] Sikorski RS, Hieter P (1989). A system of shuttle vectors and yeast host strains designed for efficient manipulation of DNA in Saccharomyces cerevisiae. Genetics.

[pgen-0030228-b066] Lisby M, Antunez de Mayolo A, Mortensen UH, Rothstein R (2003). Cell cycle-regulated centers of DNA double-strand break repair. Cell Cycle.

[pgen-0030228-b067] Smith J, Rothstein R (1999). An allele of *RFA1* suppresses *RAD52*-dependent double-strand break repair in Saccharomyces cerevisiae. Genetics.

[pgen-0030228-b068] McDonald JP, Levine AS, Woodgate R (1997). The *Saccharomyces cerevisiae RAD30* gene, a homologue of Escherichia coli dinB and umuC, is DNA damage inducible and functions in a novel error-free postreplication repair mechanism. Genetics.

[pgen-0030228-b069] Lea DE, Coulson CA (1949). The distribution in the numbers of mutants in bacterial populations. J Genet.

