# Supplementary material for: Genome-Wide Analysis of Rad52 Foci Reveals Diverse Mechanisms Impacting Recombination
Source: PLoS Genet. 2007 Dec 14;3(12):e228. doi: 10.1371/journal.pgen.0030228 (PMC2134942; doi:10.1371/journal.pgen.0030228)
Supplement: Supplementary file 1 [file pgen.0030228.sg001.pdf]

Figure S1

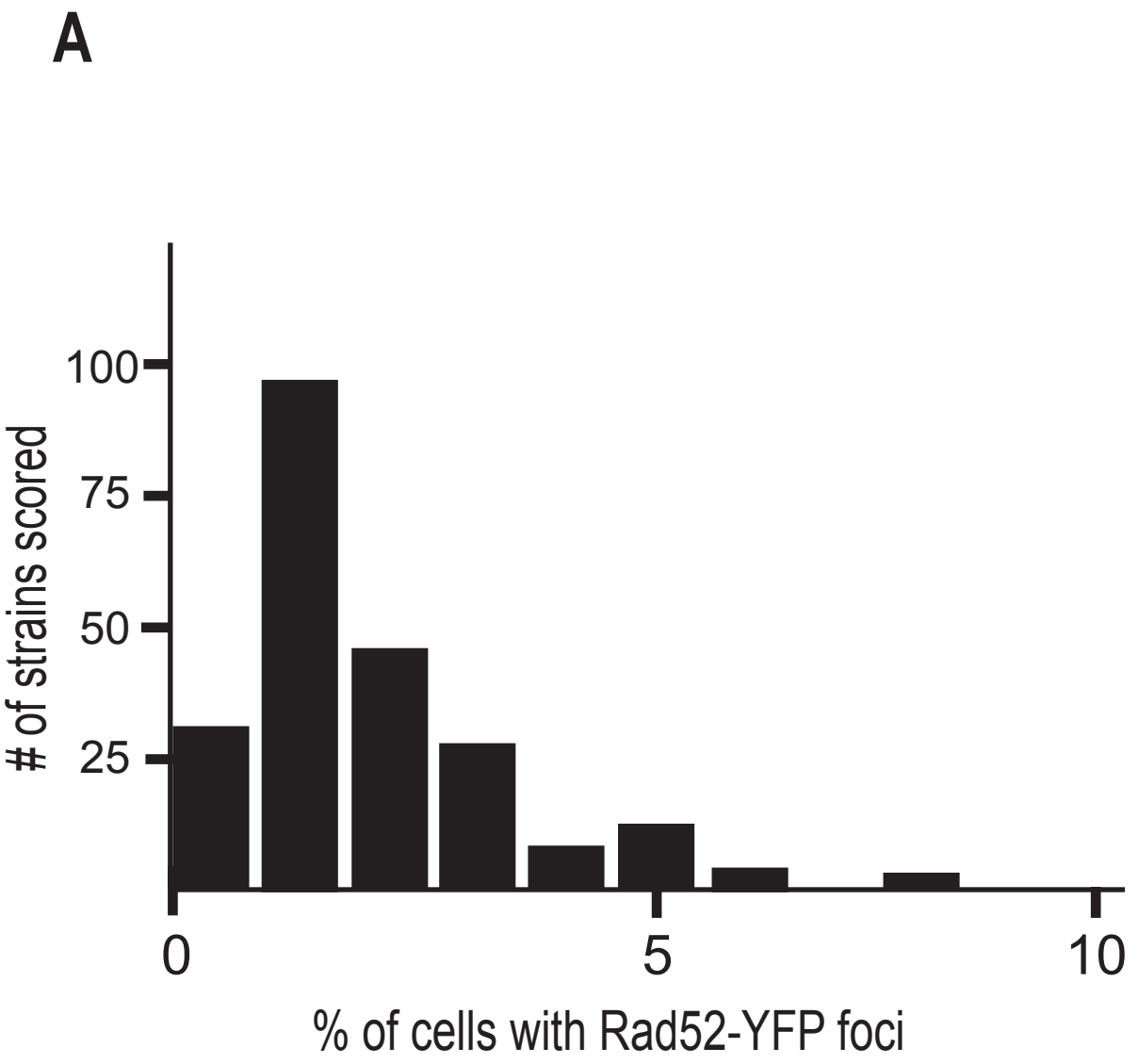

A
100
75
50
25
# of strains scored
0
5
10
% of cells with Rad52-YFP foci

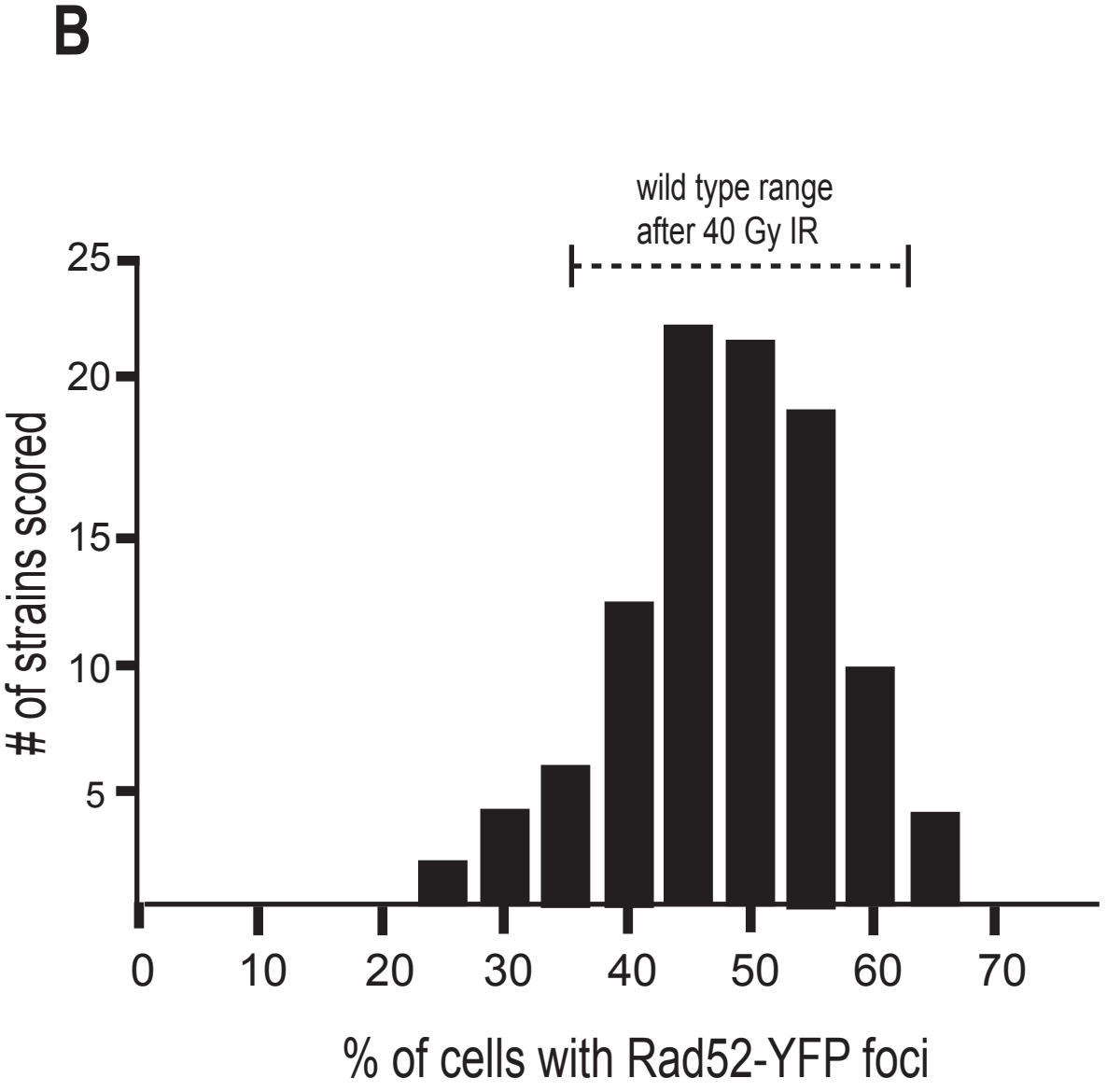

B
wild type range
after 40 Gy IR
25
20
15
10
5
# of strains scored
0
10
20
30
40
50
60
70
% of cells with Rad52-YFP foci
