# Supplementary material for: Genome-Wide Analysis of Rad52 Foci Reveals Diverse Mechanisms Impacting Recombination
Source: PLoS Genet. 2007 Dec 14;3(12):e228. doi: 10.1371/journal.pgen.0030228 (PMC2134942; doi:10.1371/journal.pgen.0030228)
Supplement: Supplementary file 2 [file pgen.0030228.sg002.pdf]

FIGURE S2

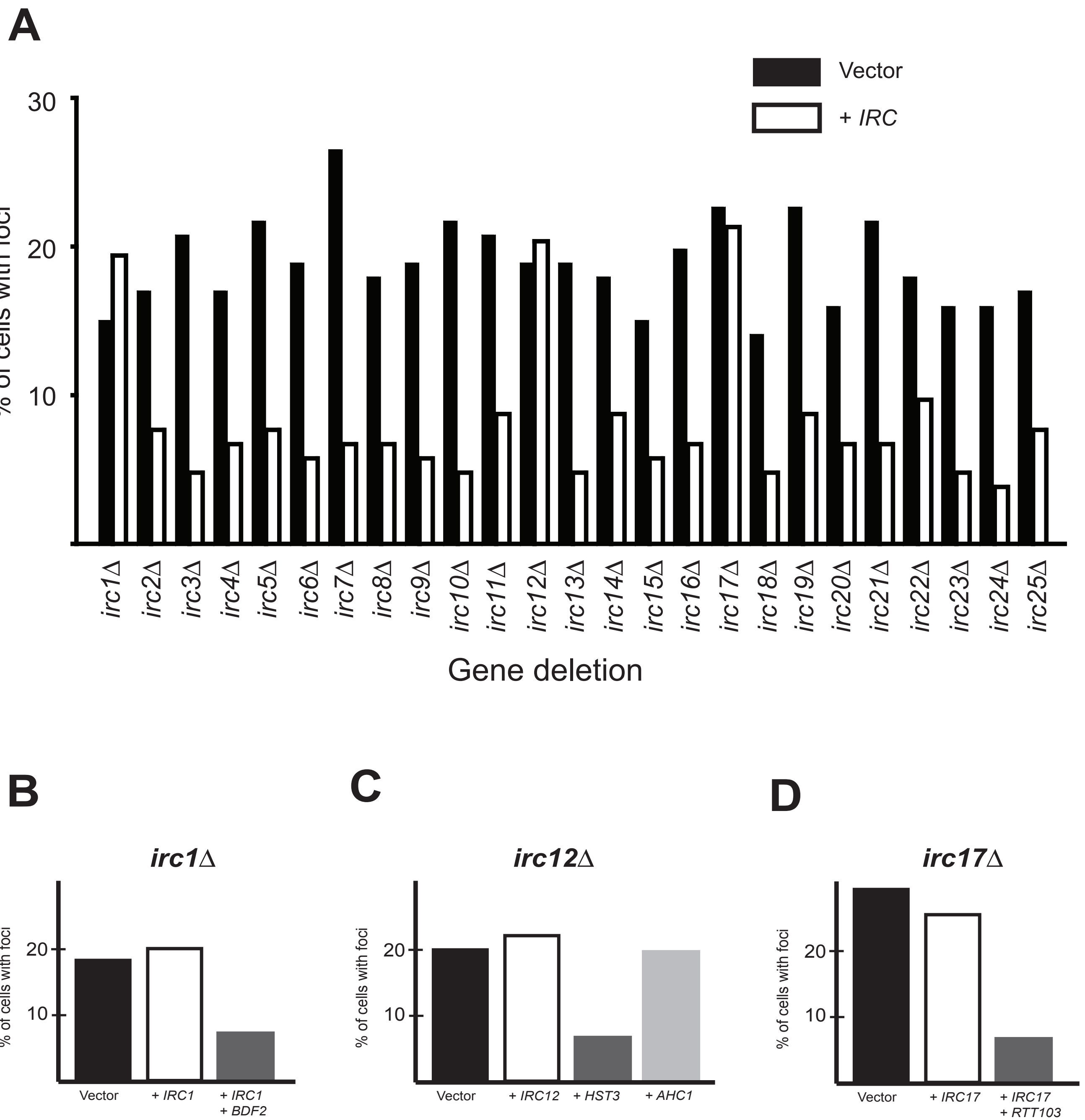
A
Vector
+ IRC
30
20
10
% of cells with foci
irc1Δ
irc2Δ
irc3Δ
irc4Δ
irc5Δ
irc6Δ
irc7Δ
irc8Δ
irc9Δ
irc10Δ
irc11Δ
irc12Δ
irc13Δ
irc14Δ
irc15Δ
irc16Δ
irc17Δ
irc18Δ
irc19Δ
irc20Δ
irc21Δ
irc22Δ
irc23Δ
irc24Δ
irc25Δ
Gene deletion
B
irc1Δ
20
10
% of cells with foci
Vector
+ IRC1
+ IRC1
+ BDF2
C
irc12Δ
20
10
% of cells with foci
Vector
+ IRC12
+ HST3
+ AHC1
D
irc17Δ
20
10
% of cells with foci
Vector
+ IRC17
+ IRC17
+ RTT103
